# High Isolation, Double-Clamped, Magnetoelectric Microelectromechanical Resonator Magnetometer

**DOI:** 10.3390/s23208626

**Published:** 2023-10-21

**Authors:** Thomas Mion, Michael J. D’Agati, Sydney Sofronici, Konrad Bussmann, Margo Staruch, Jason L. Kost, Kevin Co, Roy H. Olsson, Peter Finkel

**Affiliations:** 1 US Naval Research Laboratory, American Society for Engineering Education Postdoc, Washington, DC 02375, USA; thomas.mion.ctr@nrl.navy.mil; 2 Electrical and Systems Engineering Department, University of Pennsylvania, Philadelphia, PA 19104, USA; mdagati@seas.upenn.edu (M.J.D.); ssofroni@seas.upenn.edu (S.S.);; 3 US Naval Research Laboratory, Materials Science and Technology Division, Washington, DC 02375, USA; konrad.bussmann@nrl.navy.mil (K.B.); margo.staruch@nrl.navy.mil (M.S.); 4 US Naval Research Laboratory, Acoustics Division, Washington, DC 02375, USA; jason.kost@nrl.navy.mil; 5 Laboratoire Structures, Propriétés et Modélisation des Solides, CentraleSupélec, Université Paris-Saclay, 91190 Gif-sur-Yvette, France; kevin.co@centralesupelec.fr

**Keywords:** magnetoelectric, magnetometer, mems, iron cobalt hafnium, aluminum nitride, magnetostriction

## Abstract

Magnetoelectric (ME)-based magnetometers have garnered much attention as they boast ultra-low-power systems with a small form factor and limit of detection in the tens of picotesla. The highly sensitive and low-power electric readout from the ME sensor makes them attractive for near DC and low-frequency AC magnetic fields as platforms for continuous magnetic signature monitoring. Among multiple configurations of the current ME magnetic sensors, most rely on exploiting the mechanically resonant characteristics of a released ME microelectromechanical system (MEMS) in a heterostructure device. Through optimizing the resonant device configuration, we design and fabricate a fixed–fixed resonant beam structure with high isolation compared to previous designs operating at ~800 nW of power comprised of piezoelectric aluminum nitride (AlN) and magnetostrictive (Co_1-x_Fe_x_)-based thin films that are less susceptible to vibration while providing similar characteristics to ME-MEMS cantilever devices. In this new design of double-clamped magnetoelectric MEMS resonators, we have also utilized thin films of a new iron–cobalt–hafnium alloy (Fe_0.5_Co_0.5_)_0.92_Hf_0.08_ that provides a low-stress, high magnetostrictive material with an amorphous crystalline structure and ultra-low magnetocrystalline anisotropy. Together, the improvements of this sensor design yield a magnetic field sensitivity of 125 Hz/mT when released in a compressive state. The overall detection limit of these sensors using an electric field drive and readout are presented, and noise sources are discussed. Based on these results, design parameters for future ME MEMS field sensors are discussed.

## 1. Introduction

Magnetic field sensors have been ubiquitous in modern technology since the invention of the compass. The current state-of-the-art magnetometers have found applications ranging from biomedical fields, where high sensitivity is the crucial metric, to military sensors, where size, weight, and power (SWaP) are key factors [1,2,3]. Fluxgate magnetometers are one of the most common for naval applications as they are technologically mature, physically robust vector magnetic field sensors with sensitivities in the picoteslas at frequencies from 0.1 Hz to ~10 kHz [4]. The development of ultra-low-power miniature systems designed to supplant fluxgate magnetometers has found renewed interest in recent years [1,5].

Microelectromechanical systems (MEMS) offer an avenue of development to combine low SWaP with high sensitivity for critical systems development [6,7,8,9]. Magnetoelectric (ME)-based MEMS devices exploit the coupling occurring in artificial ME heterostructure systems by combining a piezoelectric and ferromagnetic material to produce functional devices for a multitude of engineering applications. The giant ME coupling that occurs in resonant multilayer devices has shown promise as magnetic sensors by exploiting a resonance frequency shift in response to an external magnetic field through magnetostrictive stress in the ferromagnetic material [5,10].

Magnetoelectric-based magnetometers have garnered much attention as they boast ultra-low-power systems with small form factor and limit of detection in the tens of pT [5,10,11]. Most magnetometers require tens to hundreds of mW (or more) of power to operate, such as the fluxgate, where the soft magnetic material needs to be constantly driven into a saturation state by a current source. ME-MEMS magnetometers, on the other hand, utilize a voltage source connected to a piezoelectric stack with a high impedance to drive the resonator and typically require only μW or less of power to drive the resonator, and when combined with an ultra-low-power operational amplifier for the supplemental electronics, the combined power requirements can still be sub-milliwatt. Within this class of magnetometers, multiple configurations are possible, though most rely on exploiting the mechanically resonant characteristics of a released ME heterostructure device. For example, a cantilever design is often used, which can reliably reach sensitivities in the tens of pT at low frequencies (<100 Hz); yet, when sensing a resonant magnetic field, the sensitivity can reach hundreds of fT and is theoretically only limited by the magnetic noise floor [12,13,14,15]. However, these cantilever designs are susceptible to vibrational noise, especially at low frequencies (0.1–1000 Hz), and obtaining the lowest limit of detection requires an EM shield and vibration suppression [13]. Through optimizing the resonant device configuration, it may be possible to circumvent the shortcomings of current designs. To this end, we study a fixed–fixed resonant beam structure, for it is less susceptible to vibration while providing similar characteristics to ME-MEMS cantilever devices.

Alongside the intrinsic properties of the ferromagnetic material capable of responding to an external magnetic field, the fundamental noise characteristics from accompanying electronics contribute to the limit of detection [16]. Often, the theoretical magnetic noise floor is never reached due to various noise contributions from the excitation voltage source, charge amplifier, voltage amplifier, and demodulation circuits [12,16]. Determining the inherent properties of the resonators is essential for a comparison between multiple devices and across multiple designs to decouple the resonant device from the electronics used for measurements. In an ME magnetometer sensor design, some of the most salient parameters to consider are the electric sensitivity S_el_ = $\frac{d|Y|}{df}$ (|Y| is the admittance slope between resonance and antiresonance peaks, f is the frequency), magnetic sensitivity S_mag_ = $\frac{df_{R}}{dH}$ (H is the bias magnetic field, $f_{R}$ is the resonance frequency), as well as the sensor and electronics noise floor [14]. The intrinsic sensitivity of the resonator is a product of S_mag_ and S_el_, which are measures of the resonant frequency shift per magnetic field and amplitude change per frequency shift, respectively [17]. S_mag_ values found in resonators that claim picotesla sensitivity are typically in the 50–100 Hz/mT range, while S_el_ values can vary widely depending on the specific device design and the harmonic being utilized for detection. Utilizing the fundamental resonance mode is, at times, not the most advantageous operational frequency as higher harmonics can provide larger S_el_ values and offer a lower noise floor resulting from the 1/f noise contribution. Typically, S_mag_ values are inherent to a specific resonator and, in some cases, directly related to the released stress state and, therefore, would be considered a fundamental property of the resonator, though this value ultimately can be dependent upon the resonance mode used to determine S_mag_. The sensor amplitude sensitivity can thus be defined as S_am_ = S_mag_ · S_el_, which is the intrinsic sensitivity of the magnetoelectric resonator [14,18].

In this work, we study a new design of double-clamped magnetoelectric MEMS resonators as magnetometers. Initial COMSOL^®^ modeling reveals the majority of charge generated in a fixed–fixed resonator occurs at the anchor points, leading to a conceptual redesign of the structures we previously reported [19,20,21,22]. By splitting the top electrode that runs across the surface of the beam, it is now possible to drive on one side and sense on the other. For this, we have designed the electrode to extend out a quarter of the length from both anchor points, which are then coated with a magnetostrictive material, and the remaining area on the surface to place an additional strip of the magnetostrictive material. This provides the benefit of reducing the feedthrough capacitance while maximizing the area covered by the functional magnetostrictive material. In this new design, we have also utilized thin films of a new iron–cobalt–hafnium alloy (Fe_0.5_Co_0.5_)_0.92_Hf_0.08_ that provide a low-stress, highly magnetostrictive material with an amorphous crystalline structure and ultra-low magnetocrystalline anisotropy. We have also designed a printed circuit board containing two charge amplifiers for integration of the MEMS chip for testing and evaluation.

## 2. Materials and Methods

**Figure 1:**
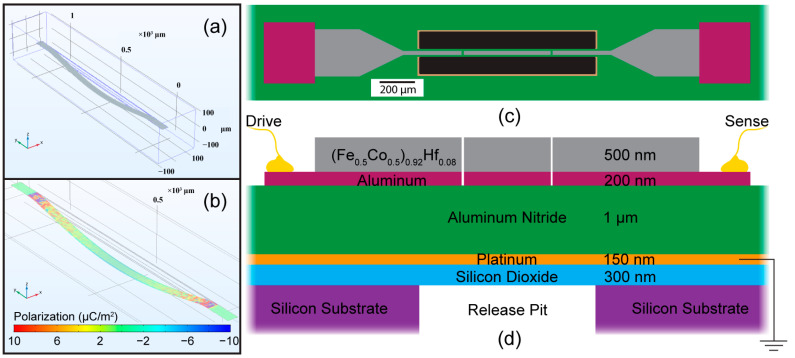
COMSOL model of (**a**) the fundamental mode (44.2 kHz) and (**b**) piezoelectric polarization generated when subjected to an external magnetic field. (**c**) Top-down view and (**d**) side view cross-section (film stack) of the suspended resonator.

COMSOL Multiphysics was used to evaluate the charge generated in a suspended double-clamped magnetoelectric resonator on which we have previously reported [20,22]. A 1 mm long and 40 um wide beam was modeled comprised of a continuous 1 μm thick aluminum nitride (AlN) piezoelectric layer mechanically coupled to a 1 μm thick layer of magnetostrictive iron cobalt (Fe_0.5_Co_0.5_) based upon our previous fixed–fixed device designs. We have previously found the fundamental frequency of the released beams is governed by the internal stresses that develop during fabrication processes. Figure 1a shows the fundamental frequency of the released resonator beam, which ranges from 30 to 40 kHz depending on the residual stresses used as input parameters in the model. Multiple stress configurations were explored during the modeling, and it was concluded that non-uniform stresses or strains applied between the piezoelectric and magnetostrictive layers cause displacements of the beam (buckling) with no applied electric or magnetic field. This results in the buckling of the beam, which is often seen in these types of suspended double-clamped structures that are not released under tension. Due to the clamped restricted boundary conditions, the resonator will buckle in the ±z direction, and when a magnetic field is artificially incorporated into the model (through stress tensor), the fundamental resonance frequency shifts higher due to the decreased buckling and effectively contributes a tensile strain on the beam. It is this shift of the resonance frequency when in the presence of a magnetic field that is the basis of detection for these types of ME sensors.

For this model, an external magnetic field is modeled by an effective change in the magnetostrictive layer, which leads to a shift in the resonant frequency. Fe_0.5_Co_0.5_ has been shown to have a saturation magnetostriction near 60 ppm; therefore, a value of 6 × 10^−6^ was used for the ε_yy_ parameter in the strain tensor, representing an effective strain resulting from the presence of a saturating magnetic field applied along the long axis of the beam. Figure 1b plots the piezoelectric polarization (surface charge) that is generated when the beam is subjected to an external magnetic field, resulting in 60 ppm strain. From the COMSOL^®^ simulations, we can see the majority of charge generated occurs at the anchor points on each end of the beam. The largest charge will always be generated by the section of the beam that is distorted away from the neutral axis within the beam. In this case, it will be at the boundaries of the resonator.

Resonators reported in this study were fabricated using the masking process developed in [23] and variations summarized in [24]. The masking steps of Ref. [23] were utilized to produce a 1 mm long and 40 μm wide suspended beam as depicted in Figure 1c. A top view of the fabricated resonators is shown in Figure 1c, where the electrically conductive materials (FeCo-Hf and Al) are sectioned into three portions. The resonator is a fully suspended bridge structure where an XeF_2_ etch process is used to release the device from the substrate with the release pit area giving substrate access to the etchant shown in black in Figure 1c. The film stack is illustrated in Figure 1d, where a (Fe_0.5_Co_0.5_)_0.92_Hf_0.08_ magnetostrictive layer provides the magnetically functional portion of the magnetometer and is deposited at 500 nm thickness. The AlN deposited was chosen to be 1 μm in order to increase piezoelectric motion of the beam, as well as to place the neutral axis of the beam at the interface between the piezoelectric and magnetostrictive material. To reduce feedthrough capacitance, the conductive top portions of the beam were separated into three sections where the drive voltage is applied to one side of the beam and charge is collected on the opposite side with the Pt bottom electrode grounded. The resonator is driven by applying an AC voltage to the AlN between the top electrode (drive) and the ground plane in Figure 1d. Subsequently, the opposite side (sense) collects the charge generated for direct readout or demodulation techniques.

**Figure 2:**
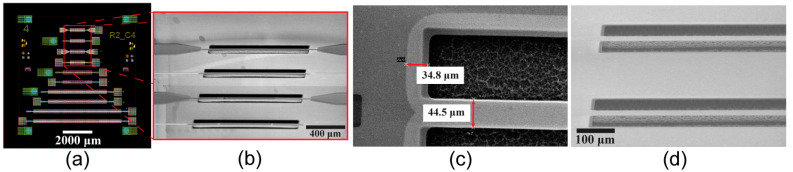
(**a**) Single 10 mm × 10 mm chip layout with multiple beam lengths and designs fabricated (**b**) tilted SEM image of four 1 mm long beams after XeF_2_ release step that were examined in this study (**c**) top-down SEM image of the beam anchor point where the lighter grey sections with red arrows show the XeF_2_ etch area and the width of the beam (**d**) high angle tilt SEM image of released beams showing the fully released suspended beams above the release pit.

Chips containing multiple beam lengths were fabricated, as seen in the combined mask image of Figure 2a. In this work, we focused on the top 4 resonators where the released length was 1 mm. A tilted scanning electron microscope SEM image of the released beams is shown in Figure 2b, where the beams can be seen buckling slightly upward. This is a sign these beams are released in a compressive stress state. To verify the beams are fully released, the undercut caused by the XeF_2_ etching process is measured, as can be seen in Figure 2c, where the light grey areas signify the undercut areas. The penetration of the SEM (30 kV) electrons allows for the contrast to be visible. Here, we can see the etching process causes ~35 μm of undercut into the Si substrate, and there is no residual Si beneath the resonator (dark gray spots), yielding a fully released beam. Figure 2d is a zoomed image of the beams where released beams show no sign of residual Si protruding beneath the beams. The presence of lingering Si fragments causes nodes in the resonator to affect the resonant properties of the beam (spurious harmonic frequencies) and consistently suppresses the motion.

**Figure 3:**
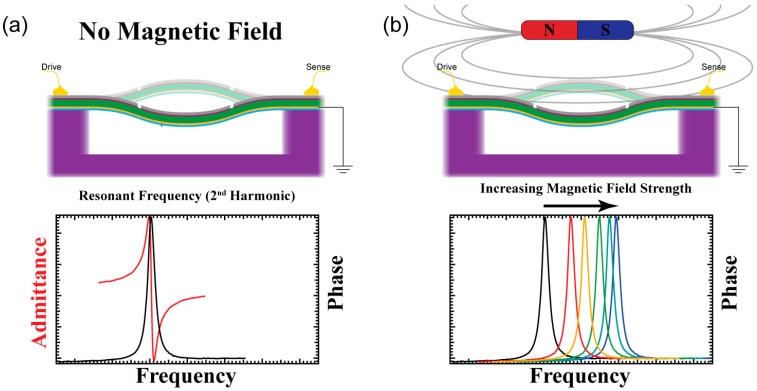
The functionality of the magnetoelectric resonator when (**a**) driven by a voltage source with no magnetic field present and (**b**) the shifting resonance frequency (phase) as a magnetic field approaches the beam (colors of phase peak plotted are not representative of field strength, only meant to signify frequency shift due to influence of a magnetic field).

Once the resonator has been released by the final XeF_2_ etch, a suspended beam is functionally complete and ready for implementation, measurement, and evaluation. The functional principle behind this type of magnetometer is a change in the resonance frequency when in the presence of an external magnetic field. Figure 3a illustrates the initial operation of the resonator, whereby one side of the beam is driven with a voltage source, and the other side senses the charge generated by the piezoelectric layer. Each beam has resonant properties (admittance and phase) associated with the mechanical impedance, as depicted in Figure 3a (when not in the presence of any magnetic field). Figure 3b shows the beam when a magnetic field is in the vicinity of the resonator, and depicted beneath is a plot of the shifting resonance frequency (phase) when subjected to an increasing magnetic field. As the magnetic field affects the magnetostrictive layer of the heterostructure, the beam expands slightly increasing the inherent buckling, thereby increasing the stress of the beam and thus shifting the resonance frequency.

**Figure 4:**
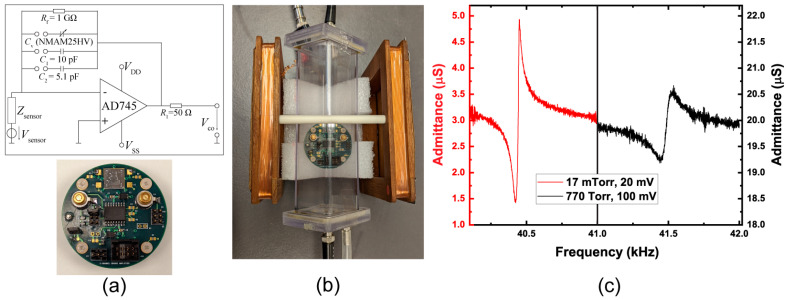
(**a**) Circuit diagram and image of charge amplifier board with sensor chip mounted. (**b**) Image of the charge amplifier placed in a vacuum chamber between coils used for applying bias magnetic field. (**c**) Admittance profile of a beam under atmosphere (770 Torr) and under vacuum (17 mTorr) plotted with the same axis scale for comparison.

The charge generated from the piezoelectric AlN element requires conditioning in order to operate as a functional sensor with a voltage output. A custom 2-channel charge amplifier board was constructed to convert the output terminals from the beams using two AD745 operational amplifiers; Analog Devices, Norwood, MA, USA. The AD745 boasts ultra-low noise performance specs of 2.9 nV/√Hz at 10 kHz, 380 nV_p-p_ for 0.1–10 Hz, and 6.9 fA/√Hz, making it a favorable operational amplifier for ME-MEMS sensors. The circuit diagram and an image of the complete charge amplifier board are shown in Figure 4a, with a magnetoelectric sensor chip mounted on the upper section of the board. The second operational amplifier is located on the underside of the PCB. When developing the charge amplifier board, we envisioned including a feedback capacitor option where a variable capacitor, C_V_, and two stable capacitors, C_1_ and C_2_, are available for selection via a jumper bank, as shown in Figure 4a, in order to adjust the gain. We ultimately believe this decision contributed to the majority source of noise, contributing to the relatively poor detection limit.

MEMS resonators physically vibrate, displacing air, and are thereby susceptible to damping by the ambient air. To reduce the damping when the resonators are placed in operation, they can be packaged under vacuum and encased in a small microchamber with a getter material to maintain a relatively low vacuum (mTorr). By operating the beam under vacuum, it becomes possible to increase the sensitivity of the magnetometer by significantly reducing the air damping [25], thereby increasing the quality factor and S_el_ value. An image of the charge amplifier board situated within a low-pressure vacuum chamber (without any wiring attached) is shown in Figure 4b. Admittance measurements of the fundamental resonance mode using a Keysight E4990A impedance analyzer (Keysight Technologies, Santa Rosa, CA, USA) of a released beam are shown in Figure 4c under atmosphere (770 Torr) and vacuum (17 mTorr). Linear fit of the slope between the series and parallel resonance peaks of the 17 mTorr scan yields a value of 2.17 × 10^−7^ S/Hz, and for 770 Torr, a value of 2.81 × 10^−8^ S/Hz represents an increase in S_el_ of 7.72-fold, with a reduction in the noise by ~1/3. The S_el_ value obtained under vacuum is similar to those found in other ME-MEMS cantilever systems in atmosphere, which tend to have much higher Q values due to the differing boundary conditions [14]. Q values of the fundamental resonance recorded in our 1 mm long devices are typically found to be ~450 in atmosphere (770 Torr), whereas under a vacuum of ~10 mTorr, the Q increases to ~1400. 

## 3. Results

### 3.1. Resonance Properties

**Figure 5:**
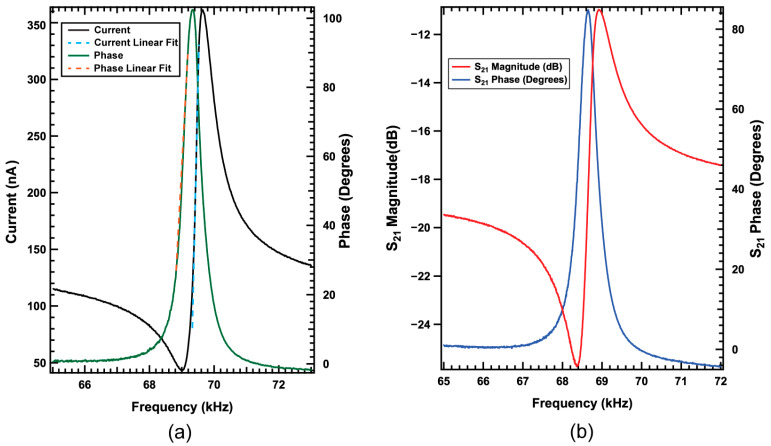
Measurements of the 2nd harmonic using (**a**) lock-in amplifier measured directly from the beam contact pads on the chip with the linear portion of the slope fitting noted by dashed lines, and (**b**) vector network analyzer after chip was mounted on the charge amplifier board, both phase plots offset to 0 for clarity.

The fundamental resonance properties depend highly on the specifics of the released beam stress state, but typically, the fundamental resonance frequency is in the 30–45 kHz range when the beams are released under compressive stresses. It was found in these beams the fundamental harmonic does not produce the highest resonance signal, and operating at the second harmonic provides a larger signal and higher bandwidth with the added benefit of operating at a higher frequency where there is less 1/f noise contribution, leading to superior sensitivity and a lower detection limit. Unless specifically noted, all data reported here were obtained using the second harmonic of the beams. Figure 5a shows the resonance profile of the beam measured directly from the contact pads on the chip using a Signal Recovery 7280 lock-in amplifier (LIA) (Ametek Scientific Instruments, Oak Ridge, TN, USA) with an excitation amplitude of 80 mV and linear fits for the measured current and phase shown by blue and orange dashed lines respectively. Linearization of the signal within the fitted area is utilized as the functional point in which to operate the sensor. Linear fit of the current resonance profile reveals a slope of 1.01 nA/Hz, a phase slope of 0.194°/Hz, and a Q of 143, as measured directly from the beam terminals. The value for Q was calculated by the typical convention by taking the resonator center frequency to bandwidth ratio. Figure 5b shows the resonance profile of the second harmonic using a Keysight E5061B ENA vector network analyzer (Keysight Technologies, Santa Rosa, CA, USA) measuring the S_21_ value of the beam when mounted on the charge amplifier board, whereby an ~80° peak phase shift shows the high-quality AlN piezoelectric properties. The operational principle underlying these types of ME-MEMS magnetometers relies on driving the resonator at a frequency within the resonance profile and monitoring either the change in the measured phase or the charge generated. The discrepancy between the reported phase values is attributed to the difference in the parasitic capacitance present in the charge amplifier board and coaxial cables used, which was seen in several sample measurements.

### 3.2. Magnetic Properties

**Figure 6:**
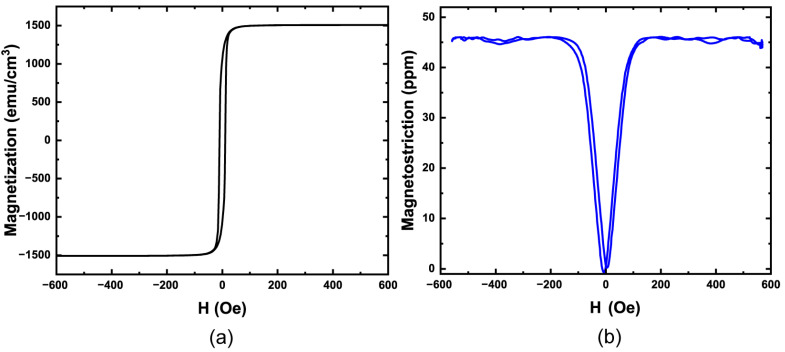
(**a**) Magnetization and (**b**) magnetostriction measurement of (Fe_0.5_Co_0.5_)_0.92_Hf_0.08_ film.

The detection of magnetic fields occurs through stress changes in the sensing element (ferromagnetic layer), and the functional property governing this stress change is magnetostriction. An alloy of (Fe_0.5_Co_0.5_)_0.92_Hf_0.08_ was developed for use in these ME-MEMS devices and found to have a low coercivity (11 Oe) while retaining high magnetization (1509 emu/cm^3^). Figure 6a shows a characteristic magnetization curve of an 18 mm diameter, 100 μm thick Si disk placed in the sputter chamber as a rider sample during deposition measured using a Lakeshore 7400 vibrating sample magnetometer (VSM) (Lakeshore Cryotronics, Westerville, OH, USA).

As discussed previously, the magnetostriction in the alloy is the functional property of the sensing element. Magnetostriction measurements of the rider sample were conducted using the cantilever method, whereby a 2 mm wide cantilever was laser-scribed from the disk and mounted between the poles of an electromagnet. The beam deflection was measured for orthogonal in-plane directions using a laser Doppler vibrometer (OFV-5000 vibrometer controller and OFV-552 fiber vibrometer) (Polytec, Hudson, MA, USA) while the applied magnetic field was swept ±1.2 kOe. Figure 6b illustrates the magnetostriction of (Fe_0.5_Co_0.5_)_0.92_Hf_0.08_, where the highest sensitivity occurs at the greatest slope on the magnetostriction curve (between 0 Oe and ±50 Oe). This alloy retains a high saturation magnetostriction (~46 ppm) with a reduced coercive field and low magnetocrystalline anisotropy than the parent compound Fe_0.5_Co_0.5_.

### 3.3. Sensor Properties and Evaluation

**Figure 7:**
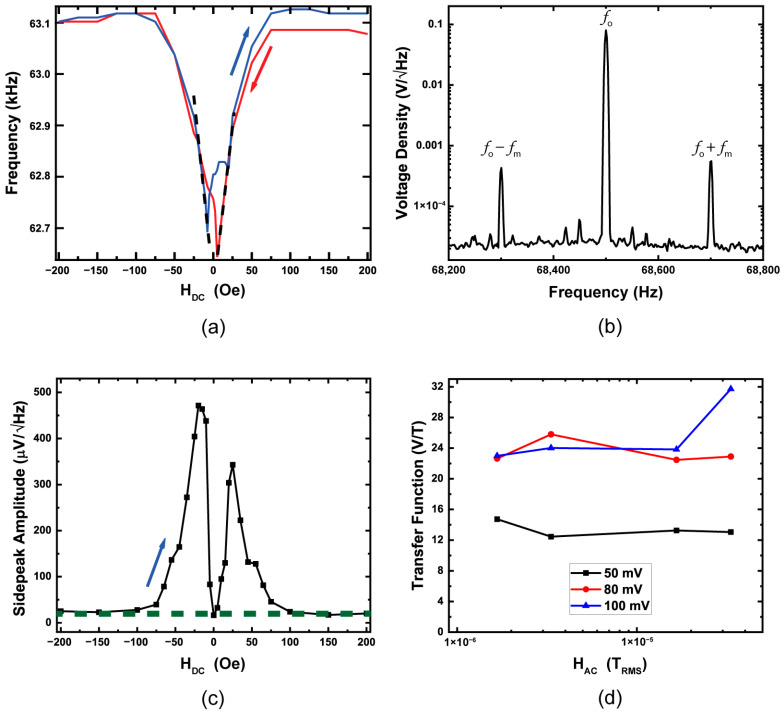
Sensor characteristics operating on the 2nd harmonic resonant frequency (**a**) magnetic bias field dependence (H_DC_) with 50 mV excitation (**b**) FFT of the modulated spectrum with H_AC_ = 11.8 μT_RMS_ and $f_{m}$ = 200 Hz (**c**) H_DC_ dependence of the sidepeak amplitudes shown in (**b**) with blue arrow indicating sweep direction (**d**) Transfer function for 50, 80 and 100 mV excitation voltages with H_DC_ = −20 Oe.

The sensitivity of the resonator relies on operating with the optimal bias magnetic field applied to the beam; this corresponds to the optimal S_mag_. To determine the ideal operational H-field, we measure the shift of the resonance frequency occurring while sweeping from a positive saturation field to a negative, and it is then reversed. For the evaluation of the sensor S_mag_, we applied the field parallel to the beam and swept to a saturation field of ±1.2 kOe. Figure 7a shows the second harmonic frequency shift as the field is swept from +1.2 to −1.2 kOe in red and the subsequent reverse sweep in blue (only ±200 Oe is shown in the figure for clarity). Initially, there is no shift of the resonance frequency due to the beam being in a saturation field, but as H_DC_ reduces to near the coercive field, a large reduction in the harmonic frequency occurs with a precipitous drop. The highest sensitivity of the beam occurs at the greatest slope, which is noted by the black dashed lines for the forward and reverse cases. From the data in Figure 7a, we can determine S_mag_ = 123 Hz/mT for the forward and 127 Hz/mT for the reverse field over a range of (±) 5 Oe to (±) 25 Oe, which makes this the optimal bias field range in which to operate the sensor. The averaged value of 125 Hz/mT is among the highest S_mag_ values reported and could be potentially increased further through post-deposition processing, such as magnetic field annealing. It should be noted the sensor can still operate with an H_DC_ up to 75 Oe, though with slightly less sensitivity. At a field strength of >75 Oe, the beam is fully in saturation and has S_mag_ = 0. While S_mag_ values are often considered an inherent property of the magnetic material response and are, therefore, representative of the device performance, it is true the bias magnetic field may affect the resonance modes differently. However, when measuring the S_mag_ values for the first three fundamental modes of an individual beam, a variance in S_mag_ ~15% was measured, and we have found this value consistent across multiple beams and chips.

Once the beam is under the optimal H_DC_ and driven at the resonance frequency, any subsequent AC magnetic field (H_AC_) will frequency modulate (FM) the drive frequency $f_{o}$. Applying H_AC_ at frequency $f_{m}$ will mix with $f_{o}$, resulting in two modulated peaks $f_{o}-f_{m}$, and $f_{o}+f_{m}$, producing modulated sidebands in the FFT of the output signal. Figure 7b shows the modulated sidebands when H_AC_ = 11.8 μT_RMS_ and $f_{m}$ = 200 Hz are applied parallel to the beam length (and parallel to H_DC_), and the resonator is driven at 50 mV measured with an SRS 780 signal analyzer (Stanford Research Systems, Sunnyvale, CA, USA). For sensitivity calculations, we use an average of both sidepeak amplitudes to yield a single representative value; the two peaks in Figure 7b provide an average of 492 μV/√Hz measured in air. The sidepeak voltage amplitude is representative of the H_AC_ strength, and the overall conversion is often called the transfer function, which, for this configuration, yields a value of 13.9 V/T.

Because the sensitivity of the resonator is dependent on S_mag_, which is, in turn, a function of H_DC_, we can track the intensity of the sidepeaks shown in Figure 7b while varying the bias magnetic field (H_DC_). Figure 7c shows the sidepeak voltage amplitude as the bias H-field is swept from a negative to positive saturation field (1.2 kOe), which is associated with the blue data line in Figure 7a. As discussed, the maximum sidepeak amplitude occurs at the highest S_mag_ (slope) in Figure 7a, and when S_mag_ = 0 (H_DC_ fields above ±75 Oe), the sidepeak amplitudes are indistinguishable from the noise floor. Additionally, the asymmetry of the slope profile in Figure 7a correlates directly with the higher peak at −20 Oe vs. +20 Oe. The green dashed line in Figure 7c represents the noise floor of measurements during the sweep, which is ~25 μV/√Hz, which also illustrates the direct correlation to S_mag_, and yet it is not unexpected since fields greater than ±75 Oe place the beams in magnetic saturation, where little or no domain motion is occurring, rendering them unresponsive.

As discussed previously, the charge amplifier output voltage is proportional to the beam excitation voltage, yet overdriving the heterostructure will produce a nonlinear duffing behavior and, subsequently, an asymmetric resonance profile. It was found these fixed–fixed resonators can be driven up to 100 mV before duffing is observed. A measure of the transfer function was performed for various excitation voltages, which is shown in Figure 7d, where a 50, 80, and 100 mV drive produces average transfer functions of 13.4, 23.4, and 25.6 V/T, respectively.

**Figure 8:**
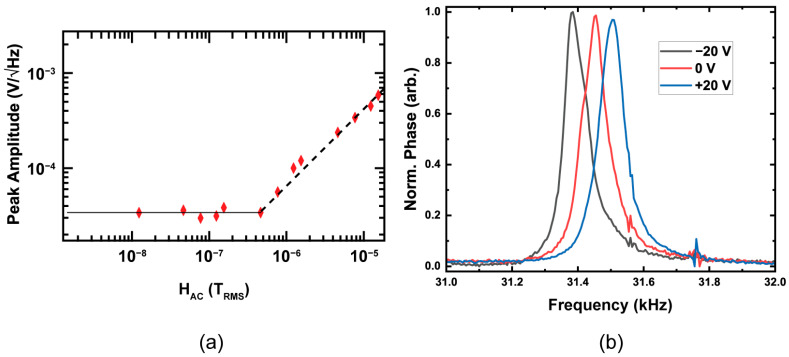
(**a**) Sensor response with H_DC_ = −20 Oe resulting in detection limit of ~460 nT_RMS_ (**b**) Bias voltage dependence of the fundamental resonance frequency.

The modulated sidepeaks visible in Figure 7b decrease with reduced H_AC_, as is plotted in Figure 8a, which shows the fitted linear response as noted by the black dashed line of a different resonator (with similar characteristics) than the one measured in Figure 7b. When the sidepeaks reduce to the noise level, as represented by the solid black line, we can extrapolate the detection limit by the intersection point. Data in Figure 8a were taken at the second harmonic with a 100 mV excitation and the beam under an optimal H_DC_ of −20 Oe, resulting in a detection limit of ~460 nT_RMS_. The noise floor for these experiments is the limiting factor, where the presence of a 25 μV noise floor prevents the detection of lower signals when measured in air. Multiple resonators were characterized using the procedures used to produce Figure 7 and Figure 8, where all showed detection limits in the 380–600 nT_RMS_ range.

Applying a DC bias electric field to the resonator can allow for tuning the resonance frequencies and improving the quality factor. Figure 8b shows the influence of applying a ±20 V bias DC voltage where a shift of a corresponding ±60 Hz is possible with an excitation voltage of 100 mV. The cumulative shift (~120 Hz) resulting from applying a 40 V DC bias would correspond to approximately the same change in stress that is the result of a change in the DC bias field ~1 mT, according to the measured S_mag_ values.

## 4. Discussion

We have demonstrated the development of a high isolation magnetoelectric MEMS resonator requiring only ~800 nW of power with an extremely small footprint capable of sensing magnetic field variations <500 nT. For these experiments, we chose an AD745 operational amplifier since it is commonly used for magnetoelectric magnetometer sensors research; it does require operation at 15 V with a quiescent current of 8 mA. This choice of operational amplifier, therefore, pulls ~120 mW of power, but other operational amplifiers are suitable replacements, such as the Analog Devices LTC6258, which only requires 105 μW, or Texas Instruments LP358 and LP2904 with a power draw of 162 μW. In this respect, we believe the potential for an ultra-low-power magnetometer is ultimately predicated on optimizing the performance of the resonator where the supplemental electronic specifications can be tailored to individual applications.

Though the ultimate limit of detection measured here is not particularly impressive, it is important to evaluate the resonator independently from the electronics and environmental noise, which are likely the main contributors to the high noise floor. Utilizing the experimental findings reported here, a comparison of detection limits is possible based on the resonator characteristics and theoretical noise floor for individual components versus the as-measured detection limits. For approximating the achievable detection limits, we will presume a resonator with the properties measured in the results section is operated under a vacuum with minimal noise contribution from the electronics. We have shown that, when under a vacuum, an increase in S_el_ of ~8× (we will define here as S_vac_) is possible and will use the measured S_mag_ of 125 Hz/mT for the calculations. Theoretical amplitude sensitivities, S_am_, of these resonators can thus be calculated from S_tot_ = S_el_ · S_vac_, yielding a value of ~27.5 μS/mT, which is consistent with other cantilever-based MEMS magnetometers [14,16,26]. Based on the noise performance of the AD745 operational amplifier when vacuum-packaged and under optimal conditions, it is possible for the resonator to reach a sensitivity of 7.2 pT when measured using current. If the resonator is measured using the voltage output generated from the charge amplifier with low-frequency demodulation, a detection limit would reach 1.66 nT and, if measured on resonance, could reach 14.7 pT. All of the theoretical limits are based solely on the noise floor of the AD745 operational amplifier, which does not consider the drive voltage noise or additional electronic as well as environmental contributions. Though these calculated detection limits may be overly optimistic, we can also estimate the detection limits of the magnetic noise and the resonator individually based on the results obtained in Section 3.

Typically, magnetometers utilizing a magnetic material and exploiting the micromagnetic structure (and subsequently domain motion) are ultimately found to have the highest sensitivity perpendicular to the magnetic easy axis. This would, therefore, suggest the highest sensitivity would occur perpendicular to the long axis of the beam and, therefore, the highest S_mag_ values. In the case of our sensors, we have found the highest S_mag_ values occur when the H-field is parallel to the long axis of the beam, which one would assume to be the easy axis of the magnetic structure. The compressive released stress state of a positively magnetostrictive material actually promotes the formation of domains to align perpendicular to the long axis of the beam. When an applied magnetic field along the long axis occurs, the domains grow and rotate nearly 90°, thus causing a large change in the resonance frequency, whereas when the field is applied perpendicularly, the domains grow but do not rotate much, resulting in a lower S_mag_ value. We measured the S_mag_ with an H-field perpendicular to the beam, which showed a significantly lower value of 26 Hz/mT. The magnetic physics of a compressively released double-clamped ME-MEMS resonator was previously analyzed and discussed in Ref. [22] as the basis for utilizing these types of resonators as vector magnetometers.

The magnetic noise of the resonator beam alone can be estimated by the intrinsic magnetic noise present and is provided by

$$b_{n}=\frac{\mu_{0}}{2}\left(\frac{dH}{df_{r}}\right)\sqrt{\frac{2\pi k_{B}Tf_{r}}{QV\sigma }}$$

where $\mu_{0}$ is the permeability of free space, $\frac{dH}{df_{r}}$ is 1/S_mag_, $k_{B}$ is the Boltzmann constant, T is absolute temperature, $f_{r}$ is the resonance frequency, Q is the mechanical quality factor, V is the resonator volume and σ is the released stress value which we can approximate to be ~50 MPa from simulation models. This would result in an intrinsic magnetic noise floor of ~9 pT/√Hz for the resonator alone and represents a magnetic noise floor far below the detection limit determined in Section 3.3. Subsequently, the only condition in which the magnetic noise floor of the resonator would become a significant factor is when the resonator is vacuum-packaged, and the accompanying electronics provide a noise floor near that of the operational amplifier specifications. Additionally, one must consider the intrinsic phase noise resulting from the compressive released double-clamped resonator. As there is an inherent correlation of the phase noise to the released stress state, maximum S_mag_, transfer function, and piezomotion, it is possible the highest values of S_mag_ contribute to a larger phase noise at the optimal bias field.

To calculate the intrinsic limit of detection achievable by the resonator using the measured values in Section 3. We can possibly utilize the calculation of voltage sensitivity from Ref. [14]. From these calculations, we find that under vacuum, with a 10 GΩ feedback capacitor in the charge amplifier, we can obtain a detection limit of ~780 pT. When calculating this limit of detection, we assume a noise level of 1 μV, which is a realistic noise level stemming from all the charge amplifier components. Ultimately, we believe the true limit of detection for these resonators to be in the 500 pT to 1 nT regime with an improved charge amplifier and accompanying electronics.

Future measurements intended to extract the lowest noise floor possible will include performing the experiments in a shielded environment with an ultra-low noise excitation voltage source with a voltage subtraction circuit included. Additionally, the resonators will be incorporated into a vacuum-packaged device with the presence of larger flux concentrators applied parallel to the beam length for additional sensitivity.

## Data Availability

The data that support the findings of this study are available from the corresponding author upon reasonable request.
